# Supplementary material for: Synthesis and Antibiotic Activity of Chitosan-Based Comb-like Co-Polypeptides
Source: Mar Drugs. 2023 Apr 15;21(4):243. doi: 10.3390/md21040243 (PMC10143536; doi:10.3390/md21040243)
Supplement: Supplementary file 1 [file marinedrugs-21-00243-s001.zip › marinedrugs-2302712-supplementary.pdf]

marine drugs

MDPI

Supplementary Information

Synthesis and antibiotic activity of Chitosan-based comb-like co-polypeptides

Timothy Enright[1], Dominic Garcia[1], Gia Storti[1], Jason Heindl[2], and Alexander Sidorenko[1,*]

MTT assay for cell viability

The cytotoxicity of CHI-g-Polypeptides was evaluated against Human Dermal Fibroblasts (HDF) cells using a MTT (3-[4,5-dimethylthiazol-2-yl]-2,5-diphenyltetrazolium bromide) assay. MTT is reduced in the mitochondria of metabolically active cells by succinate dehydrogenase to yield formazan, a water-insoluble crystal which is strongly colored purple. The amount of formazan is proportional to the number of viable cells and is quantified spectroscopically. Following a method adopted from Riss *et al.* [1]. Cells were grown in Sigma-Aldrich's Fibroblast Growth Medium and seeded to a concentration 10^5 cells/well in a microtiter plate and incubated for 24 h at 37 °C in 5 % CO_2. Next, increasing amounts of test compounds were added to each well and again incubated for 24 h. MTT was then added to a concentration of 0.45 mg/ml and incubated. After 3 h DMSO was added to dissolve any formazan crystals and absorbance was measured at 570 nm.

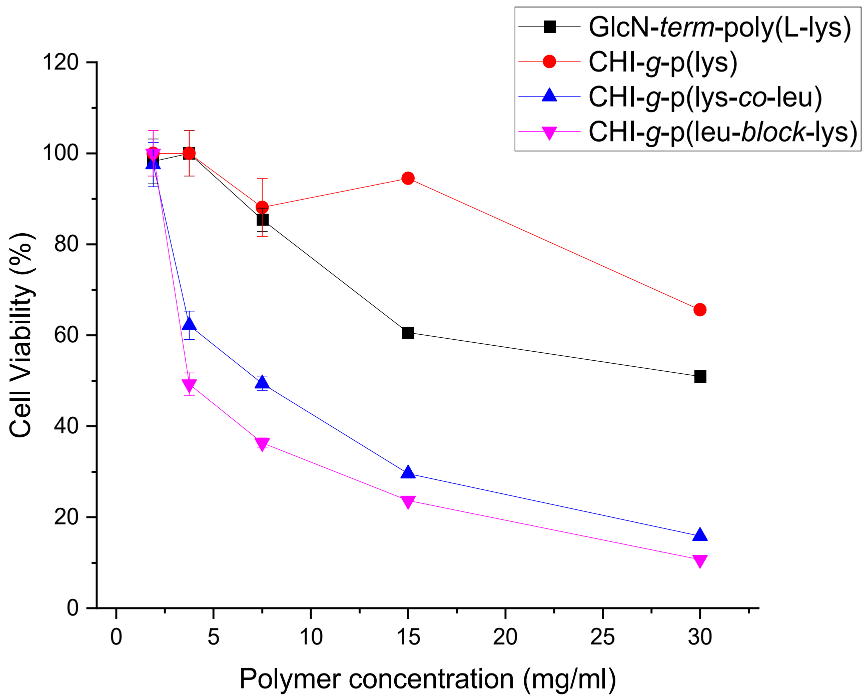

Figure S1. MTT cell viability assay results. Human Dermal Fibroblast (HDF) cells were exposed to the indicated concentrations of glucosamine-terminated poly(lysine) (black), CHI-graft-poly(lysine) (red), Chi-graft-poly(lysine-co-leucine) (blue), and Chi-graft-poly (leucine-block-lysine) (magenta) for 24hrs. Data are presented as the mean ± standard deviation, n=3.

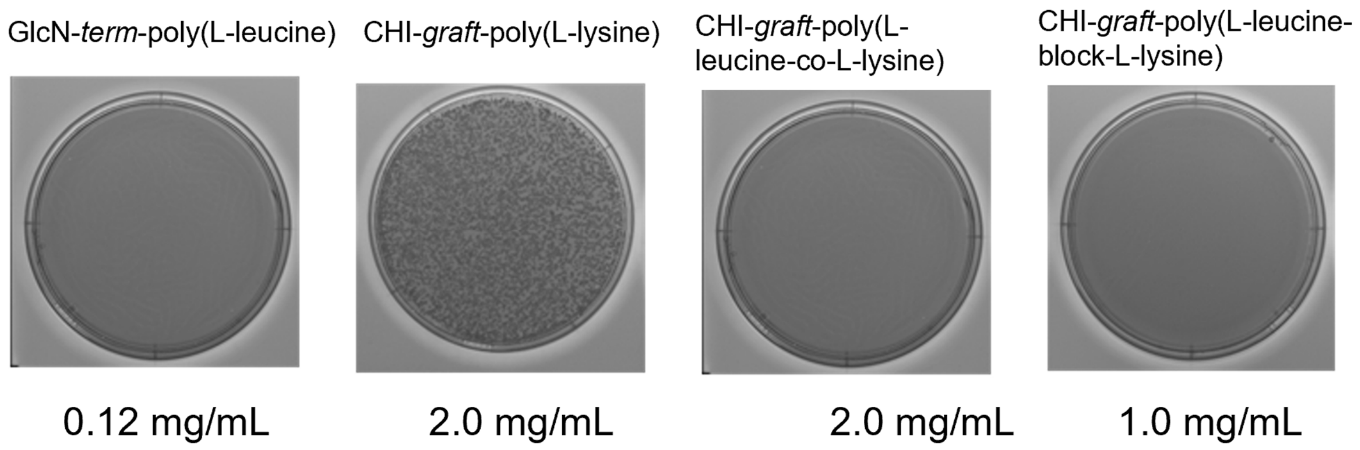

Figure S2. MBC Plates. *E. coli* cultures with the lowest concentration of graft copolymer that exhibited no change in growth were subcultured to determine minimum bactericidal concentrations. Each image displays bacteria growth on Mueller-Hinton agar plates after 24 h incubation. Note 2.0 mg/mL was the highest concentration tested and is below the MIC of CHI-*graft*-poly(L-lysine), i.e. MIC of CHI-*graft*-poly(L-lysine) is not reached at the concentration as high as 2.0 mg/mL.

Reference

S1. Riss, T.L.; Moravec, R.; Niles, A.; Duellman, S.; Benink, H.; Worzella, T.; Minor, L. Cell viability assays assay guidance manual. *Assay Guid. Man.* **2004**, 1–23.
